# Acute Fatty Liver of Pregnancy: An Obstetric Emergency

**DOI:** 10.7759/cureus.111239

**Published:** 2026-06-21

**Authors:** Umm E Amara, Umme Nashrah, Nissar Shaikh, Firdous Ummunnisa

**Affiliations:** 1 Obstetrics and Gynaecology, Deccan College of Medical Sciences, Hyderabad, IND; 2 Medicine, Deccan College of Medical Sciences, Hyderabad, IND; 3 Surgical Intensive Care, Hamad Medical Corporation, Doha, QAT; 4 Obstetrics and Gynaecology, Dr. Halima Al Tamimi, Obstetrics and Gynaecology Centre, Doha, QAT

**Keywords:** abdominal compartment syndrome, acute fatty liver of pregnancy, fulminant hepatic failure, hemorrhage, hepatic encephalopathy, multiple organ failure, transfusion-related acute lung injury

## Abstract

Acute fatty liver of pregnancy (AFLP) is a rare but serious hepatic disorder associated with significant maternal morbidity and mortality. While hepatic encephalopathy and multiorgan failure are recognized complications, the occurrence of transfusion-related acute lung injury (TRALI) and abdominal compartment syndrome (ACS) in the context of AFLP is rarely reported. We present a case of AFLP complicated by fulminant hepatic failure, hepatic encephalopathy, TRALI, ACS, and multiorgan dysfunction. A young primigravida at 36 weeks of gestation presented to the emergency department with nausea, vomiting, and jaundice. An immediate lower uterine segment caesarean delivery was performed, resulting in the birth of a live male infant. Postoperatively, the patient exhibited progressive deterioration in neurological status, worsening hepatic function, and acute kidney injury, necessitating admission to the surgical intensive care unit. Infectious and metabolic causes of hepatic failure were excluded, and viral hepatitis serologies were negative. She received intensive resuscitation with blood products, intravenous dextrose, and N-acetylcysteine. Abdominal haemorrhage, TRALI, and ACS complicated her clinical course. She progressed to fulminant hepatic failure with associated hypoglycaemia, hypoproteinaemia, hepatic encephalopathy, and ultimately brain herniation. AFLP remains a potentially life-threatening obstetrical emergency. Our patient had two risk factors for AFLP as a primigravida and carrying a male foetus. She had multiple organ failures in addition to TRALI and ACS, leading to a fatal outcome.

## Introduction

Acute fatty liver of pregnancy (AFLP) is a rare but potentially life-threatening obstetric emergency, often resulting in not only hepatic failure but also multisystem organ dysfunction [1]. Although previously considered universally fatal, early recognition and timely intervention have significantly improved outcomes. Recent literature estimates maternal mortality rates between 7% and 18% and foetal mortality rates ranging from 9% to 23% [2]. A prospective study reported the incidence of AFLP to be approximately five per 100,000 deliveries [3]. While AFLP commonly presents with hepatic and renal dysfunction, complications, such as transfusion-related acute lung injury (TRALI), are rarely described. Furthermore, only two cases of AFLP complicated by abdominal compartment syndrome (ACS) have been reported in the literature.

We report a fatal case of AFLP complicated by fulminant hepatic failure, multiorgan dysfunction, TRALI, and ACS.

## Case presentation

A 23-year-old Syrian primigravida at 36 weeks of gestation presented to the emergency department with a two-day history of diarrhoea, nausea, and vomiting. She had no past history of medical, surgical, or obstetric disorders, no abortion, and was not on any medications. She was a housewife with normal dietary habits and lifestyle. Initial evaluation revealed acute kidney injury and hepatic dysfunction. A working diagnosis of HELLP syndrome (hemolysis, elevated liver enzymes, and low platelets) or pregnancy-related liver dysfunction was made. An immediate lower uterine section caesarean delivery (LSCS) was performed under general anaesthesia. General anaesthesia was selected due to the presence of coagulopathy and increased bleeding risk, making neuraxial techniques such as spinal anaesthesia contraindicated due to the potential for spinal epidural hematoma.

Standard ASA (American Society of Anaesthesiology) monitoring was employed, including continuous electrocardiography, non-invasive blood pressure measurement, pulse oximetry, and capnography. Invasive arterial blood pressure monitoring was also established. The estimated blood loss was approximately 800 mL, managed with fluid resuscitation and transfusion of blood products as required. A viable male neonate was delivered, and maternal resuscitation was continued postoperatively.

On the first postoperative day, the patient exhibited clinical deterioration characterized by confusion, tachycardia (heart rate: 120 bpm), tachypnoea (respiratory rate: 20-26 breaths per minute), and hypothermia (35.2°C), while maintaining stable blood pressure. Laboratory investigations revealed anaemia (hemoglobin: 7.9 g/dL), normal white blood cell count, and a normal platelet count (204,000/µL), but there was active oozing from the surgical site. Coagulation studies were significantly deranged, and both total and direct bilirubin levels were elevated. The patient also experienced an episode of hypoglycemia and showed evidence of worsening renal function, although urine output remained preserved. Abdominal ultrasonography demonstrated hepatic steatosis consistent with fatty liver (Figure 1).

**Figure 1:**
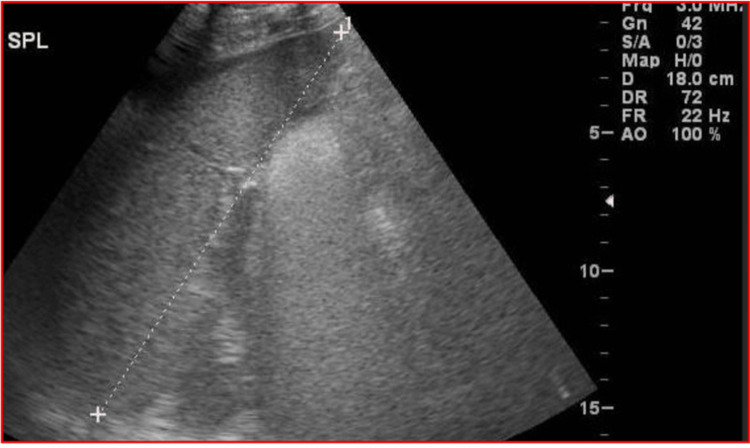
Ultrasonography showing bright fatty liver There is diffuse increased echogenicity of the liver (“bright liver” appearance).The liver appears more echogenic than the renal cortex.

The patient was transferred to the surgical intensive care unit (SICU) for ongoing critical care. Management included continued hemodynamic resuscitation with blood products, initiation of empiric broad-spectrum antibiotics, 10% dextrose infusion for hypoglycaemia, and intravenous N-acetylcysteine to support hepatic function. Despite these measures, her clinical status worsened with progressive hepatic dysfunction, persistent hypoglycaemic episodes, worsening coagulopathy, and new-onset thrombocytopenia (Tables 1, 2). Over the course of her SICU stay, she received a total of 12 units of packed red blood cells (pRBCs), 12 units of fresh frozen plasma (FFP), and 18 units of platelet concentrates.

**Table 1:** Coagulation profile The results show thrombocytopenia, prolonged prothrombin time (PT), international normalized ratio (INR), activated partial thromboplastin time (aPTT), and low fibrinogen levels.

Day	Platelets (10^3^/uL)	Prothrombin time (seconds)	INR (international normalized ratio)	Activated prothrombin time (seconds)	Fibrinogen (grams/L)
0	253	45.6	3.9	75.4	8
1	106	83.6	7.3	80.56	0.1
2	90	25.6	2.4	37.4	1.2
3	74	26.6	2.5	34.6	1.2
4	60	28.8	2.9	35.2	1
5	24	21.6	2.2	37.7	1.3
6	40	11.6	1.5	31	1
7	34	19.1	1.8	32.1	0.8
8	32	19.2	1.5	31.4	1.2
9	44	17.2	1.6	33.3	1.6
10	36	17.9	1.4	35.5	2.2
11	26	16.6	1.6	42	2.3
12	33	16.9	1.6	51	2
13	33	17.9	1.6	43	2.1

**Table 2:** Liver function profile The results show lactatemia, low albumin, and abnormal liver function.

Day	Lactic acid (µMol/L)	Albumin (grams/L)	RBS (mmol/L)	Total bilirubin (Umol/L)	AST (units/L)	ALT (units/L)	Alkaline phosphatase (units/L)	Total Protein [Grams/L]
0	11.2	24	3.2	152	470	328	422	44
1	4.6	20	1.9	162	265	211	337	35
2	3.2	22	1.8	188	154	118	189	41
3	3.7	24	2.4	221	108	78	134	44
4	3.4	27	1.6	267	79	53	110	45
5	4.6	29	1.4	249	43	32	78	50
6	5.04	26	1.2	288	55	30	71	45
7	5.25	30	2.1	412	73	31	87	41
8	2.98	27	2.9	372	66	34	98	42
9	3.1	26	3.6	397	67	45	111	41
10	2.32	24	3.4	339	70	65	136	31
11	4.1	23	3.1	394	99	56	176	34
12	5.1	25	3.2	455	85	57	305	41
13	4.2	26	2.9	504	110	76	471	40

On day 3, the patient’s condition acutely deteriorated. She became disoriented and agitated, with oxygen saturation dropping to 78%, necessitating endotracheal intubation and mechanical ventilation. Chest radiography revealed bilateral diffuse pulmonary infiltrates. Central venous pressure remained within normal limits, and the partial pressure of oxygen to fraction inspired oxygen (P/F) ratio was <300. In the context of massive transfusion of blood and blood products, the clinical picture was consistent with TRALI (Figure 2).

**Figure 2:**
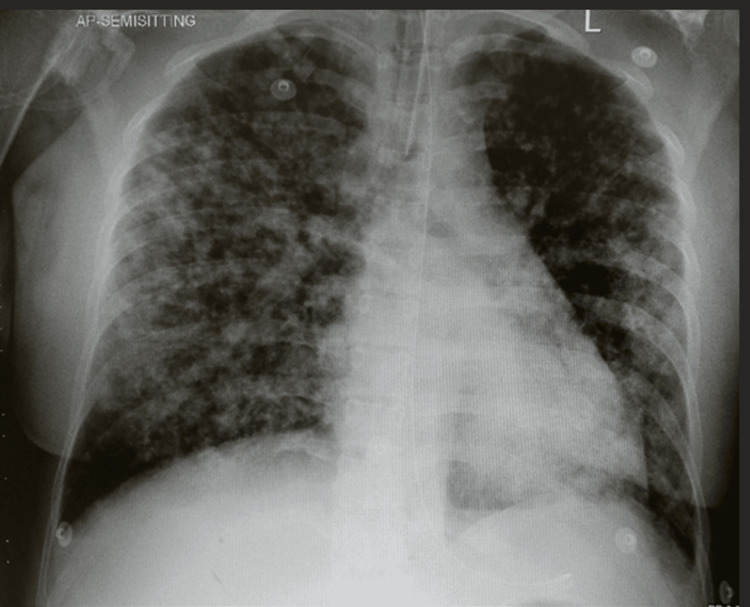
Chest X-ray showing diffuse bilateral infiltrates Chest radiograph demonstrates diffuse bilateral pulmonary infiltrates/opacities involving both lung fields.

The liver function continued to decline, with marked elevations in transaminases and serum ammonia. A non-contrast CT brain scan demonstrated cerebral edema (Figure 3), which was managed with intravenous hypertonic saline (3% NaCl), strict fluid management, head-of-bed elevation, and neuroprotective supportive measures. Despite these interventions, her neurological status continued to worsen, consistent with evolving hepatic encephalopathy and fulminant liver failure.

**Figure 3:**
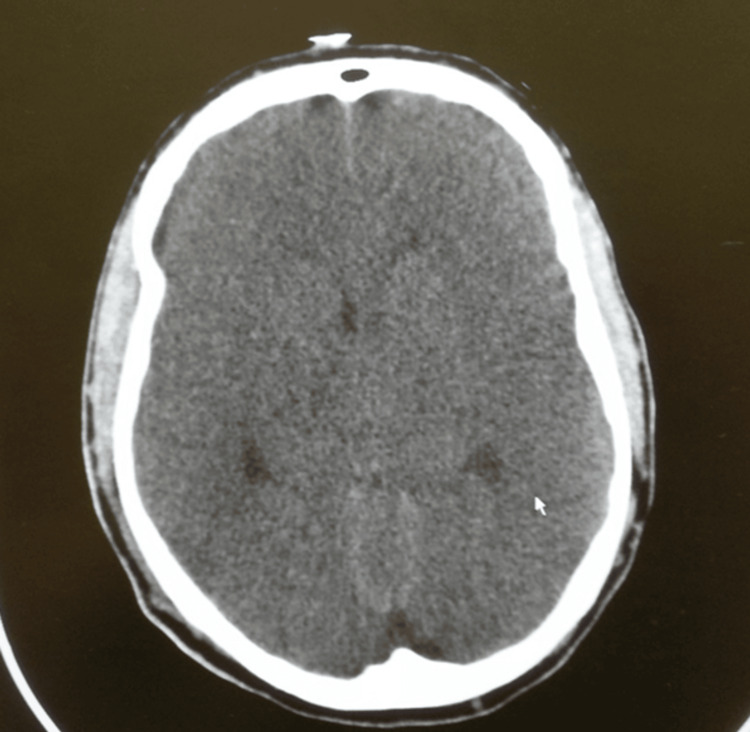
CT brain showing diffuse brain oedema Non-contrast CT brain demonstrates diffuse cerebral edema characterized by loss of normal gray-white matter differentiation.

On day 5, the patient developed progressive abdominal distension with a measured intra-abdominal pressure of 23 mmHg, reduced urine output, and worsening respiratory mechanics requiring escalation of ventilator support. Bedside abdominal ultrasonography revealed intraperitoneal free fluid. A diagnosis of ACS was made, and the patient was taken emergently for exploratory laparotomy. Intraoperatively, she required norepinephrine infusion and ongoing resuscitation with blood and blood products. Findings included a rectus sheath hematoma and approximately two liters of fresh and clotted blood within the peritoneal cavity.

On day 6, she experienced a tonic-clonic seizure, managed initially with benzodiazepines and subsequently started on levetiracetam. CT imaging of the brain revealed diffuse cerebral oedema with signs of impending herniation. Mannitol was added to her therapy. Despite all intensive resuscitative efforts, her condition continued to deteriorate with worsening liver dysfunction and coagulopathy (Tables 1, 2). On day 7, her pupils became bilaterally dilated and non-reactive. Despite full supportive measures, she progressed to multiorgan failure and was pronounced dead on day 9.

Informed consent for participation and publication was obtained from the patient’s family.

## Discussion

Liver dysfunction complicates up to 3% of pregnancies and, although relatively uncommon, may rapidly progress to fulminant hepatic failure with substantial maternal morbidity and mortality [4]. AFLP, previously referred to as acute yellow atrophy of the liver, is considered an obstetric emergency. Prompt delivery combined with intensive supportive care remains the cornerstone of management [5]. The reported incidence of AFLP varies; a prospective study estimated it to occur in approximately five per 100,000 pregnancies [3]. Clinical presentation is variable, ranging from nonspecific gastrointestinal symptoms, such as nausea and vomiting (64%) and diarrhea (16%), to fulminant hepatic failure (>70%) and renal dysfunction (>50%) [5].

Our patient also reported a history of diarrhea and presented with both hepatic and renal dysfunction. The diagnosis of AFLP is primarily clinical, supported by imaging and the Swansea criteria, and is made after excluding other potential causes [6]. The Swansea criteria are used to diagnose AFLP. A diagnosis of AFLP is confirmed if six or more of the following 15 clinical, laboratory, and radiographic features are present and unexplained by other medical conditions [6]. It is essential to distinguish AFLP from other hepatic disorders in pregnancy, particularly viral hepatitis, as the management strategies differ significantly [7]. In this case, all hepatitis serologies were negative, and other inherited metabolic disorders known to cause hepatic failure were systematically excluded. AFLP must be differentiated from HELLP syndrome as AFLP primarily involves severe liver failure and clotting abnormalities, while HELLP is characterized by high blood pressure, red blood cell destruction, and low platelets [8].

AFLP is a disorder of mitochondrial fatty acid oxidation, characterized by impaired metabolism of long-chain fatty acids due to a deficiency of the mitochondrial enzyme long-chain 3-hydroxyacyl-CoA dehydrogenase (LCHAD). This enzymatic defect leads to the accumulation of fat droplets within hepatocytes, resulting in microvesicular steatosis. Risk factors associated with AFLP include preeclampsia, primigravida status, multifetal gestation (especially twin pregnancies), and carrying a male fetus [5]. Our patient had two recognized risk factors: she was a primigravida and carrying a male fetus.

AFLP is associated with multisystem involvement and a wide range of complications. Reported manifestations include fulminant hepatic failure (54%), hypoproteinaemia (75%), hemorrhage (43%), acute kidney injury (39%), disseminated intravascular coagulation (32%), and hepatic encephalopathy (20%) [5]. Fatal complications are typically characterized by markedly elevated bilirubin, prolonged prothrombin time, increased INR, and decreased fibrinogen levels [5]. Unfortunately, our patient exhibited all of these high-risk features. In addition, she developed persistent hypoglycaemia despite glucose supplementation and progressed to hepatic encephalopathy. Notably, she also developed TRALI (defined by new-onset, non-cardiogenic pulmonary edema and acute hypoxemia developing during or within six hours of a blood and fresh frozen plasma transfusion and ACS (diagnosed by intra- abdominal hypertension of more than 20 mmHg and recent two organ dysfunction) - complications that have not been widely reported in association with AFLP. While AFLP was once considered nearly universally fatal, more recent retrospective data suggest significantly improved outcomes, with one study reporting a mortality rate of just 7%, possibly attributed to the inclusion of patients without severe complications [5].

Prompt management after early diagnosis of AFLP is critical to improving maternal outcomes. Most of the literature supports immediate termination of pregnancy, most often via caesarean delivery, as delays or attempts at vaginal delivery are associated with increased maternal mortality [9]. Immediate delivery of the fetus is the cornerstone of the management of AFLP; the mode of delivery should be individualized according to the maternal and foetal condition. Early delivery interrupts the pathological accumulation of fatty acids in the liver by eliminating the placenta, which plays a central role in maternal fatty acid metabolism. Patients with multiorgan dysfunction require intensive care support. In our case, intravenous N-acetylcysteine was administered for hepatic protection, as suggested in previous reports [10].

To conclude, our patient developed multiorgan failure, including fulminant hepatic failure and hepatic encephalopathy, complicated by severe haemorrhage requiring massive transfusion. This, in turn, contributed to the development of TRALI and ACS. The combination of these critical complications ultimately led to maternal mortality in this case.

## Conclusions

Early recognition and prompt delivery of the fetus is critical to improving maternal outcomes. Our patient had risk factors to develop AFLP; unfortunately, she developed multiorgan failure, including fulminant hepatic failure and hepatic encephalopathy, complicated by severe hemorrhage requiring massive transfusion. This, in turn, contributed to the development of TRALI and ACS. The combination of these critical complications ultimately led to maternal mortality in this case. The AFLP patients can develop into ACS and TRALI, coagulopathy, and bleeding, which have a negative impact on patient outcome.
